# Self-management system for postpartum women with hypertension disorders: an eHealth application intervention study

**DOI:** 10.1186/s12884-023-05483-y

**Published:** 2023-03-16

**Authors:** Chung-Wei Chang, Yi-Jing Tsai, Yu-Yun Hsu, Ting-Wei Hou

**Affiliations:** 1 Department of Engineering Science, National Cheng Kung University, Tainan, Taiwan; 2 Department of Nursing, College of Medicine, National Cheng Kung University, Tainan, Taiwan

**Keywords:** Postnatal care, Postpartum hypertension, eHealth, Multi-platform, Mobile devices

## Abstract

**Background:**

Hypertension disorders are relatively common in pregnant women and often persist in the postpartum period. Few studies are available regarding the self-management of postpartum hypertension via the eHealth system. This study aimed to develop a self-management eHealth system for women with postpartum hypertension during the postpartum period.

**Methods:**

We adopted a multi-platform system for this research, not only for use on the web interface but also on smartphones. The proposed system possessed three features: (1) the population was limited to postnatal women with hypertension; (2) a self-care record, which allowed postnatal women to keep track of their blood pressure, pulse, weight, medication record, exercise record, and risk factor assessment; and (3) through this system, nurse-midwives could keep track of postnatal women’s health status maintaining the complete record and could communicate directly with the users if their health monitor values reach beyond normal range.

**Results:**

Thirty-nine postnatal women with postpartum hypertension were recruited to the study. A survey to evaluate the usability and satisfaction of the proposed e-health application system was completed by these women. The usability rate of the system reached 92.4% (46.2% satisfied and 46.2% strongly satisfied), which suggested that the system was helpful to the users. The satisfaction rate of the system reached 94.9% (43.6% satisfied and 51.3% strongly satisfied), which suggested that the system was acceptable to the users.

**Conclusion:**

This proposed system has been developed completely with user experience and professional advice from experts. Postnatal women could gain important postpartum-related knowledge and access their related health records and other information easily via their smartphones or computers. During the postpartum period, an eHealth application system can effectively assist women with hypertension to manage their blood pressure and related postnatal healthcare issues.

## Introduction

Hypertension disorders of pregnancy are a common pregnancy disease that affects the health of pregnant women and their babies. Hypertension disorders of pregnancy are classified into four types: preeclampsia, gestational hypertension, superimposed preeclampsia, and chronic hypertension [1]. According to the Japan Society for the Study of Hypertension in Pregnancy (JSSHP), superimposed preeclampsia is defined as hypertension accompanied by organ damage or proteinuria. Preeclampsia is defined as hypertension occurring after 20 gestational weeks along with proteinuria, organ damage, or uteroplacental dysfunction. Gestational hypertension is defined as hypertension alone after 20 gestational weeks [1, 2].

Hypertension disorders of pregnancy account for about 5–7% of cases (such as preeclampsia, which is a major cause of preterm birth and an early marker for future cardiovascular and metabolic diseases) and it is the third most common cause of maternal mortality and fetal morbidity during the perinatal period [3, 4]. A study revealed that 10% of maternal deaths are attributed to a hypertension disorder of pregnancy in the postpartum period [5]. The majority of maternal and neonatal mortality occurs during the early postpartum period [6]. A focus on postnatal management is increasing, reflecting increasing evidence of risk of recurrence of hypertensive disorders of pregnancy in subsequent pregnancies [7].

Postpartum is the most neglected time for the provision of quality services [8]. Lack of appropriate care during this period could result in significant ill health and even death [9]. Therefore, it was suggested that postpartum women should monitor their blood pressure daily and maintain good health behaviors to promote their self-health and avoid or reduce the risk of postpartum hypertension. With an expansion in mobile environments along with other eHealth technologies, opportunities for eHealth are increasing [10–13]. Mobile Apps for postnatal care are rarely available in the App market. However, some Apps were designed specifically for baby care and not for postpartum women with special needs such as postpartum hypertension.

Hence, the main purpose of this study was to develop an eHealth self-management application system for postnatal women with hypertension after birth, to keep track of their health status through the advice of nursing professionals. The main functions of this system were as follows:

- Multi-platform and with an ability to run on various devices.
- A graphical display of daily blood pressure, pulse, and weight changes, inclusive of the recommended reasonable range of blood pressure, pulse, and weight, with the intention of making it easier for the users to read.
- The graphical display of blood pressure with a statistical pie chart included that showed 7 to 90 days of data and people could choose their own date range.
- A live version of an exercise instructional video, which postnatal women could follow and take part in the exercises.
- A medication record management for postnatal women who were required to take medication to control hypertension.
- A risk factor assessment, using a simple health scale to let users know their status.
- A smart chat room, where postnatal women could ask questions, and the system had built-in some questions that could be answered automatically and smartly.
- A postnatal related health education information for postnatal women which could be updated at any time.

## Methods

### System design

**Fig. 1:**
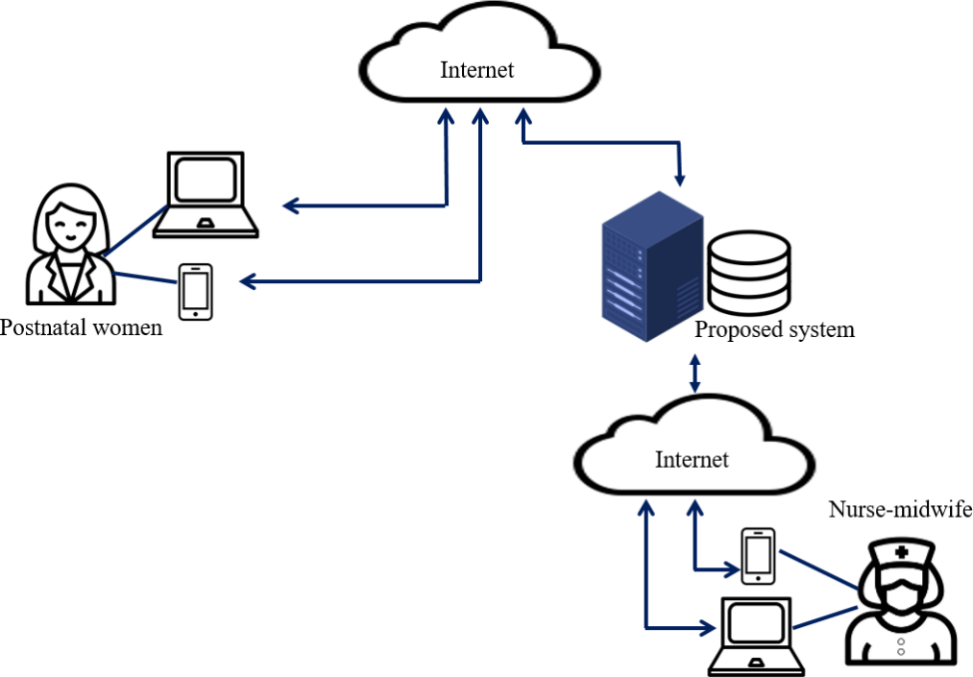
The architecture of proposed system

The architecture of the proposed system is presented in Fig. 1. This proposed system was composed of internet-connected smartphones and computers. Every postnatal woman could use her smartphone or computer to connect to the proposed system via internet services and use the various functions provided by the system. Postnatal women were only permitted to access and manage their own information when they logged in to the system. An authorized nurse-midwife could access and manage the information of all postnatal women.

**Fig. 2:**
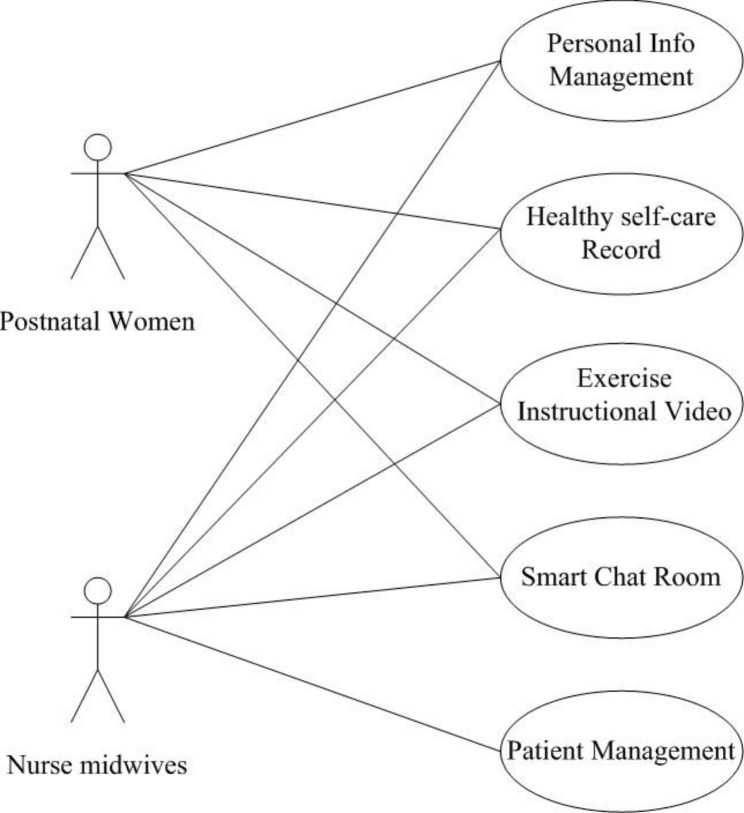
Use case diagram

A user case diagram of the proposed system is presented in Fig. 2. Postnatal women were able to add, retrieve and edit personal information, and access healthy self-care records, the exercise instructional video and the smart chat room. Nurse-midwives were able to access all of the functions of the system, and they were authorized to retrieve and manage all the information about the postnatal women under their care, for example, the users’ personal information, and health self-care records.

**Fig. 3:**
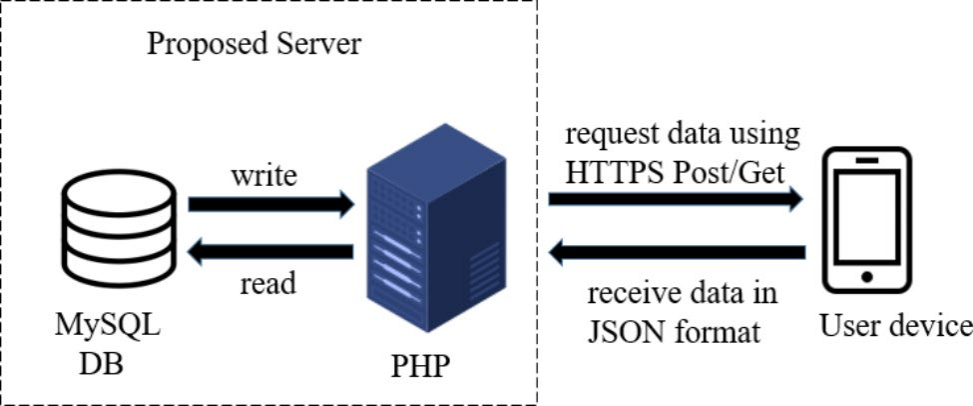
Exchange of the data between the proposed server and the user device

Figure 3 shows how the data are retrieved from the database in the proposed server. A PHP class was written to get data from and store data in the database. This would encode the retrieved data into the JSON [14] format and send it to a Multi-platform device. The application then would parse the JSON format data and displays it on the Multi-platform device.

### Implementation

The proposed system is written in PHP7 [15] and HTML5 [16] and run on a remote server with an Apache HTTP Server 2.4. Users can access the system using web browsers on their smartphones or computers. MySQL database is used in the system.

Data security is very important to internet systems. HTTPS is used to establish an encrypted link between the server and the client. We use OpenSSL [17] for secure transfers, which is an open-source implementation of the secure sockets layer (SSL v2/v3) and transport layer security (TLS 1.0/1.1/1.2) protocols.

**Fig. 4:**
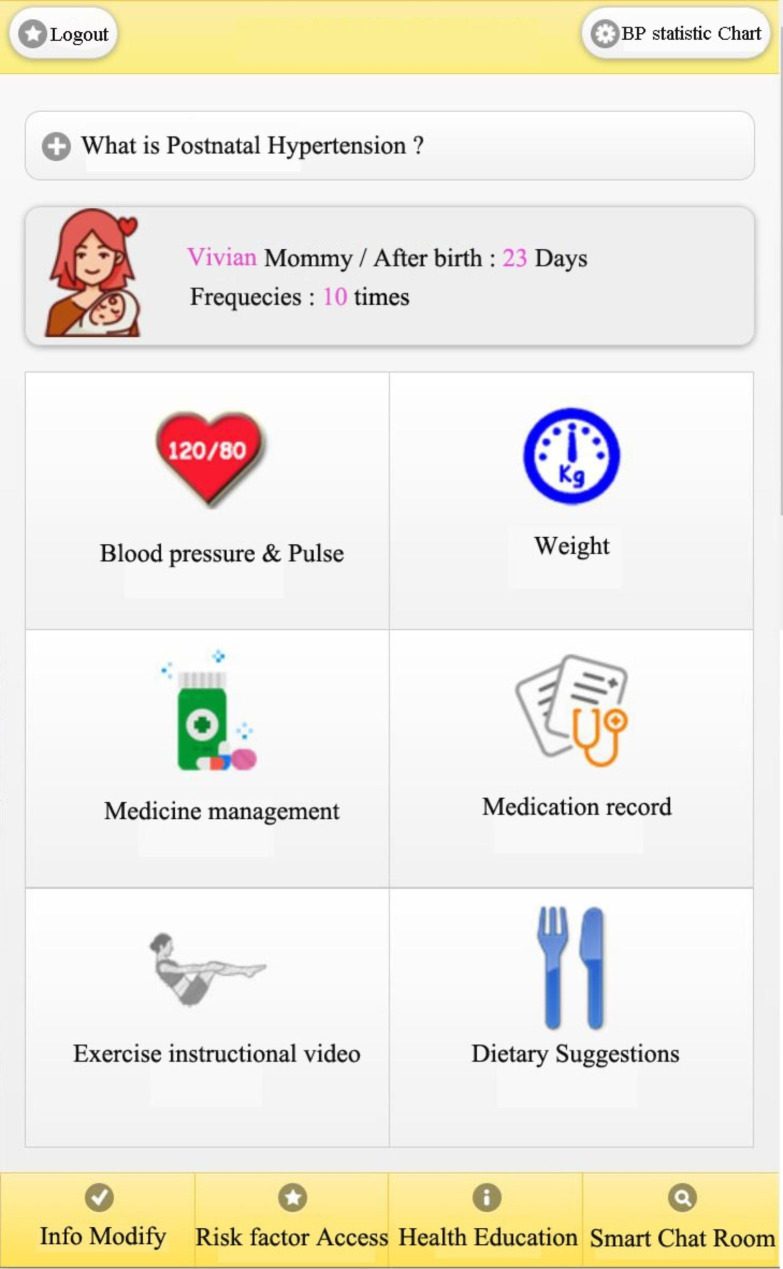
Main Page

Regarding the system, there is a main page for user login, as shown in Fig. 4. After logging in, the top of the page displays the number of days of the postnatal period, and the login frequencies of users. Each function on this control panel is introduced and explained.

**Fig. 5:**
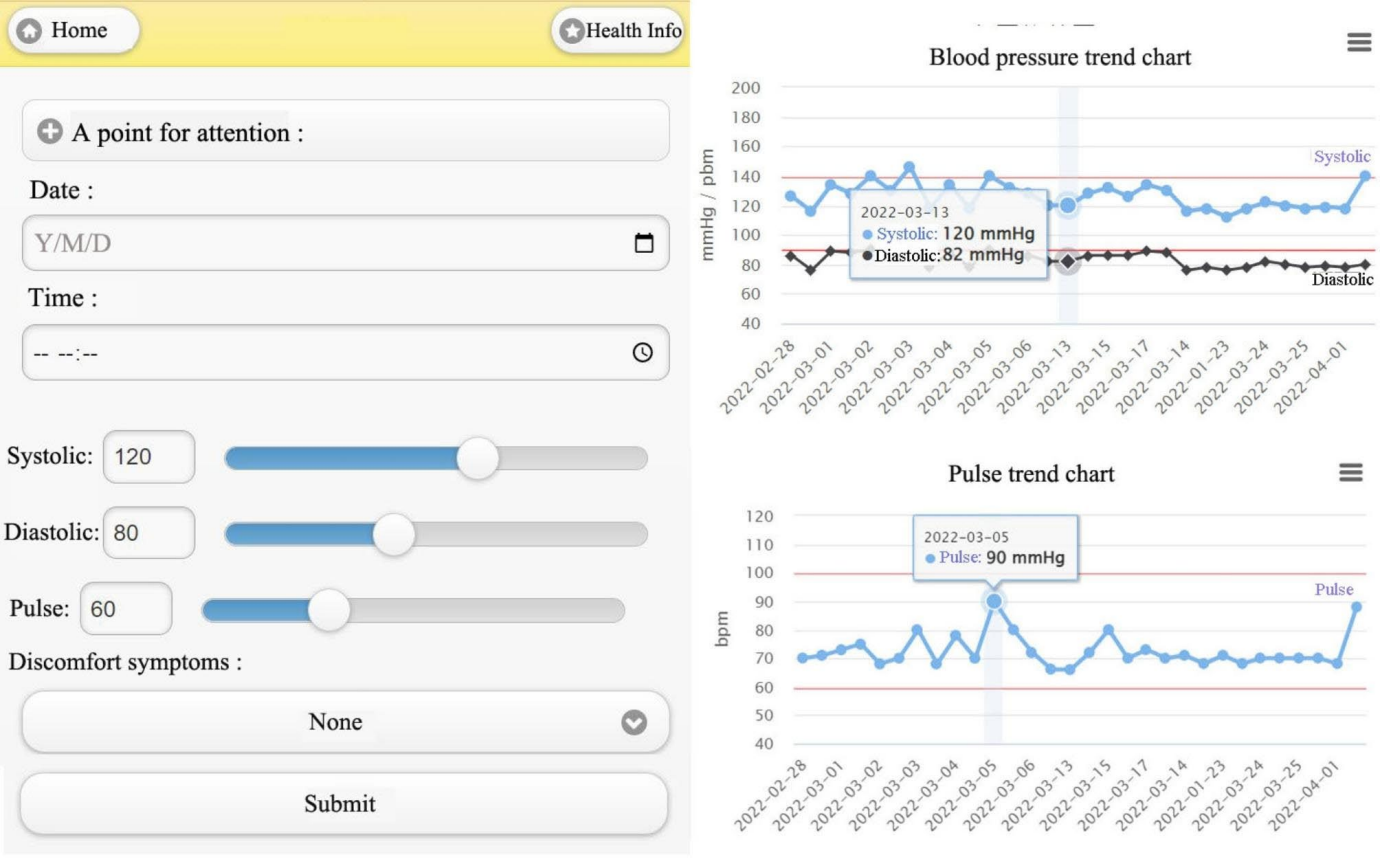
Blood pressure and pulse record

In the blood pressure and pulse record section, postnatal women need to enter the date and time, systolic and diastolic records, and pulse records, as shown in Fig. 5. The right side of the system displays a line graph [18] and each time an entry is made, the entry is recorded as a point in the graph. The upper limit of systolic pressure (140 mmHg) and diastolic pressure (90 mmHg) are indicated by the red line, and the user can check whether the red line is exceeded or not. A second graph presents the pulse records where the upper limit of pulse (100 bpm) and the lower limit of pulse (60 bpm) are indicated by the red line, and the user can check if this line is exceeded or not.

The system assesses three blood pressure states: (i) whether the systolic pressure (less than 120 mmHg) and the diastolic pressure (less than 80 mmHg) are normal, (ii) a systolic pressure greater than 120 mmHg but less than 139 mmHg or diastolic pressure greater than 80 mmHg but less than 89 mmHg which are warning ranges and (iii) a systolic pressure greater than 140 mmHg or diastolic pressure greater than 90 mmHg and if this persists for a few days or not. If the blood pressure has not improved, then the user is advised to attend the hospital directly to see the obstetrician.

**Fig. 6:**
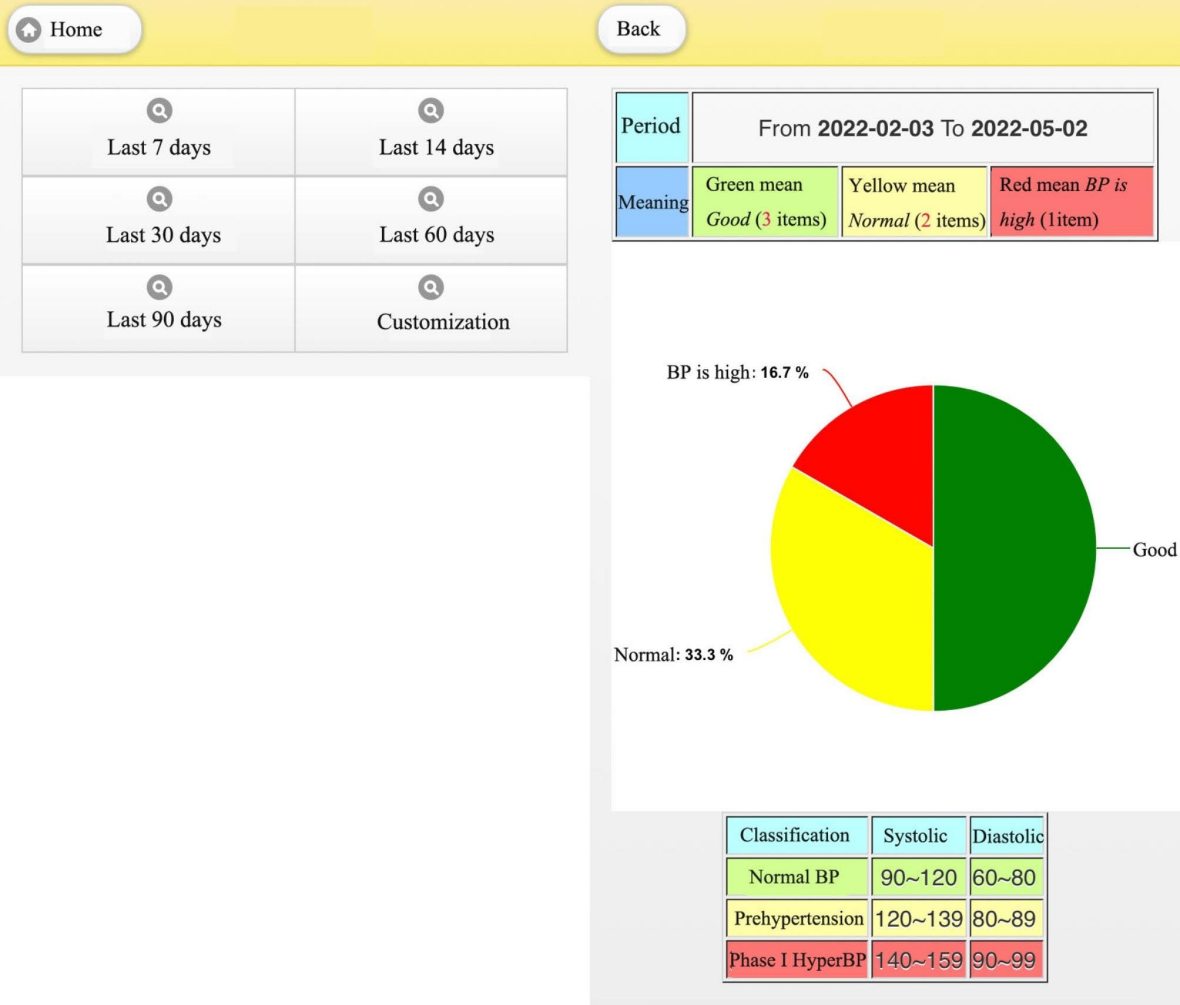
Pie chart of blood pressure measurement

In the blood pressure pie chart [18] postnatal women can choose the appropriate period, like 7 to 90 days of data or a customized date range, as shown in Fig. 6. These statistics are indicated by different colors: green means good, yellow means normal and red means high blood pressure. The regular return visit should be 6 weeks after birth for natural births, 10–12 days and 6 weeks after the birth for caesarean section births. The blood pressure statistics can be provided to the doctor to evaluate the postpartum blood pressure and adjust a woman’s medication if required.

**Fig. 7:**
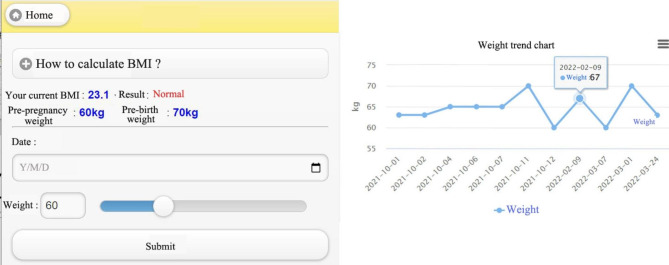
Weight record

In the section on body weight, postnatal women need to enter their daily weight records, as shown in Fig. 7. In general, postnatal women would return to their pre-pregnancy weight 6th week after the birth. BMI is used as a reference to assess the weight change before as well as throughout the pregnancy.

**Fig. 8:**
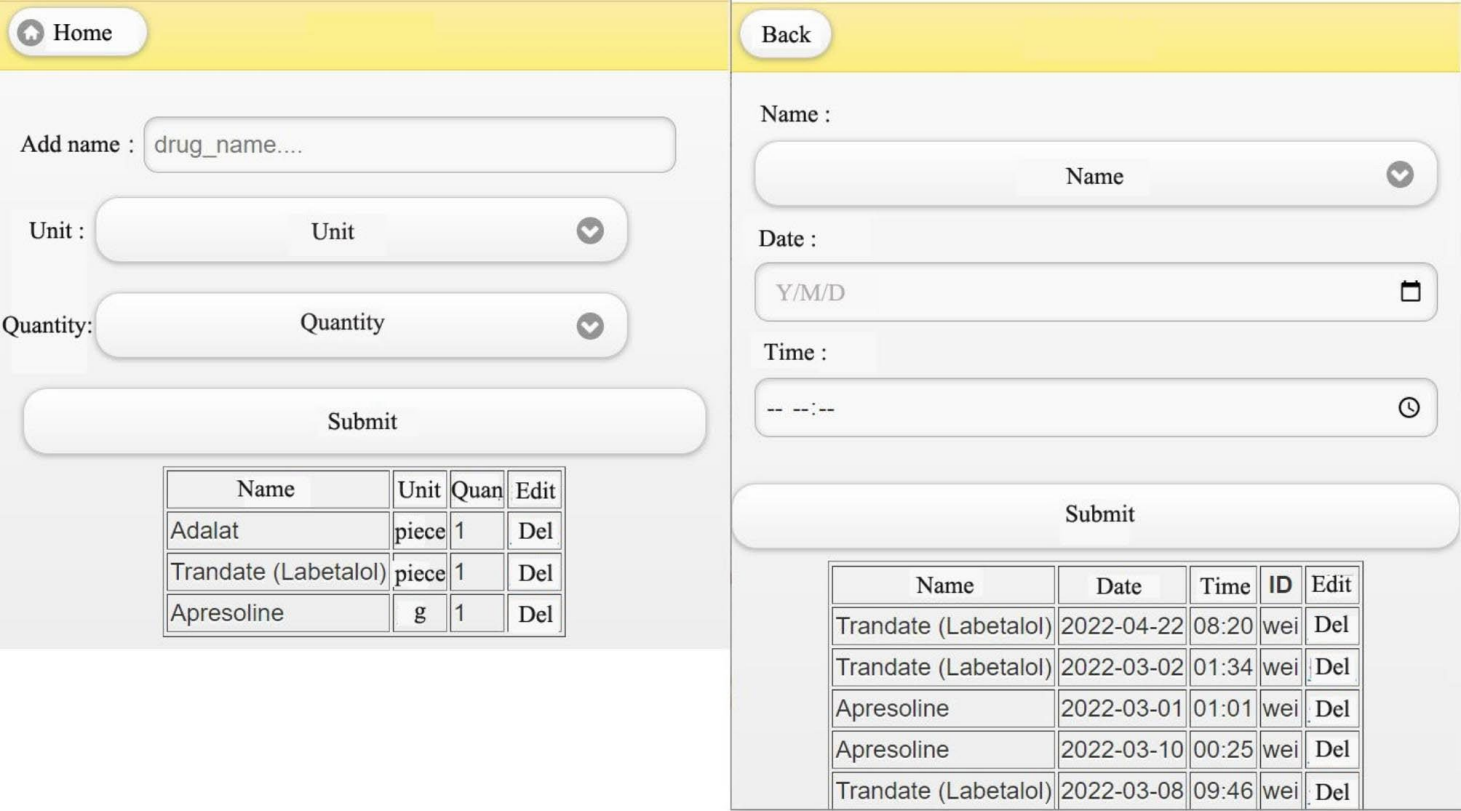
Medication record

The user’s medication management is shown in Fig. 8. Medication management, displayed on the left side of the system includes some built-in anti-hypertensive drugs (Fig. 8). If a woman is taking drugs not built into the system, she could add these (right side, Fig. 8). On the right side is the medication record, and users can make a record when taking medication by entering the name of the medication, the date of use and the time of use, and use the calendar function as a medication reminder. The bottom of the display page shows the detailed record of each medication.

**Fig. 9:**
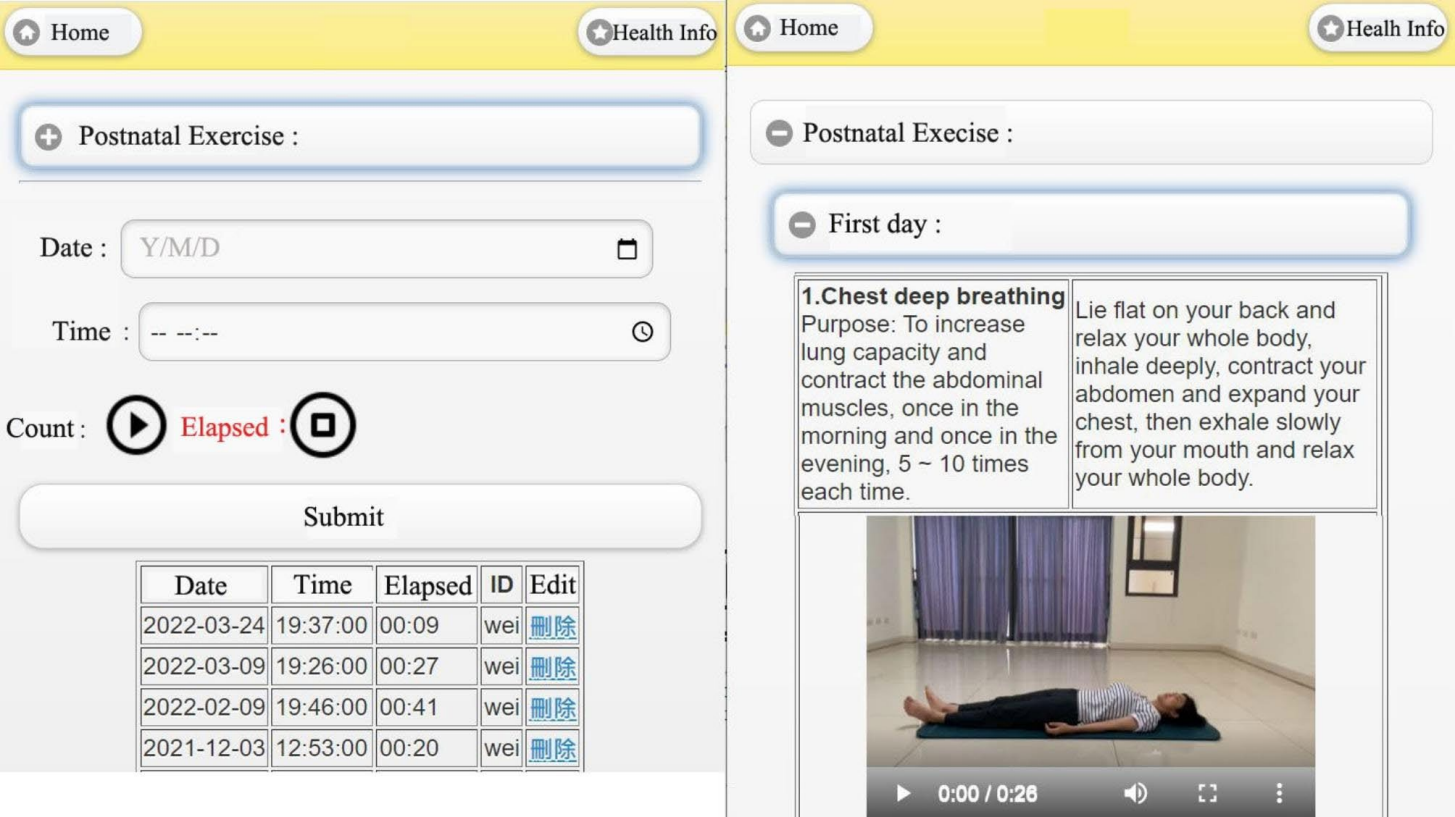
Exercise instructional video

Figure 9 shows the exercise instructional video [19]. On the right side is the introduction to the postnatal exercises. There are suitable exercises for postnatal women from the first day to the 15th day after birth. Through the live demonstration video, users can follow along with the video exercise, and background voice instructions can help women undertake the exercises correctly and efficiently. The instructions start with simple and easy-to-use exercises. Initially, there is only one type of exercise, and additional exercises are then gradually added one by one every day. On the left side of the display is the record of exercise time. Users can select an exercise with an associated video, select the date and time period, and then start the exercise by clicking the play button to start the time. Once the exercise is over, users can click the end button, then a record of the exercise will be completed.

**Fig. 10:**
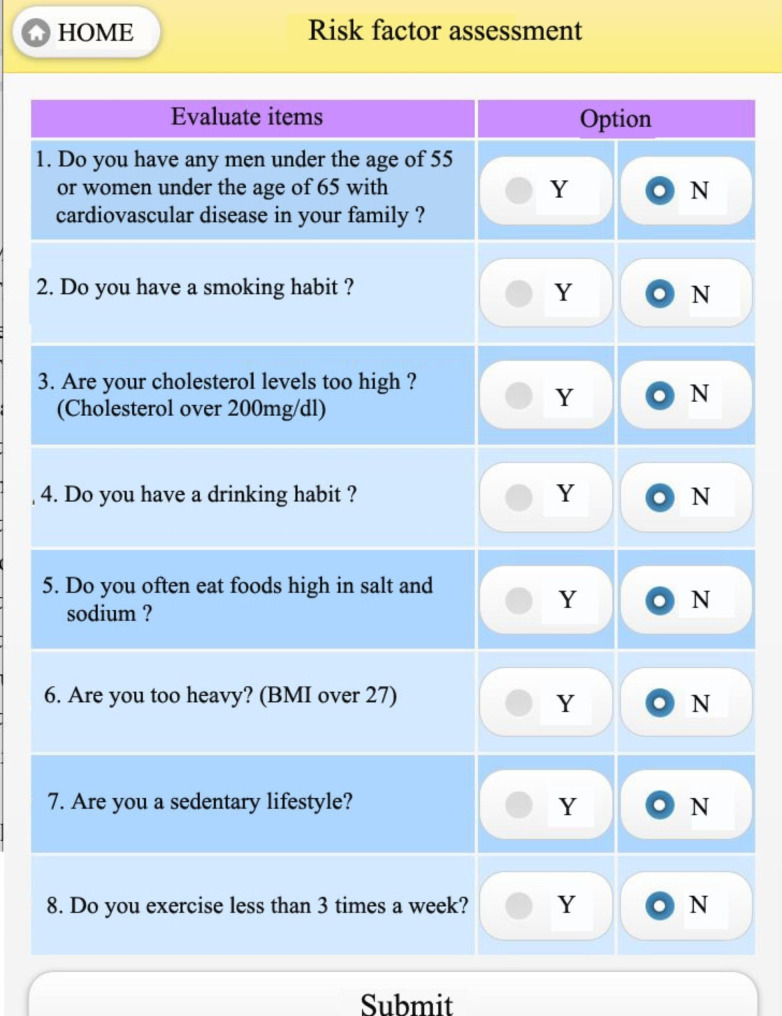
Assessment of risk factors

The assessment of risk factors [20] is shown in Fig. 10. There are 8 risk factors for hypertension listed in this assessment form. It is known that hypertension can be caused by several factors such as heredity, smoking habits, excessive body weight, excessive sodium intake, and insufficient exercise. So, an evaluation is carried out to analyze how many risk factors the user has. It is noteworthy to mention that the more risk factors a woman has then the more likely she is to experience hypertension. The system thus provides a simple assessment: if a woman ticked ‘yes’ to more than 3 risk factors, this was an alarm signal and warranted attention; if a woman indicated ‘yes’ for more than 5 risk factors, this was a cause for serious concern for the health of the woman. If a woman had several (3 or more) risk factors efforts should be made to reduce these.

**Fig. 11:**
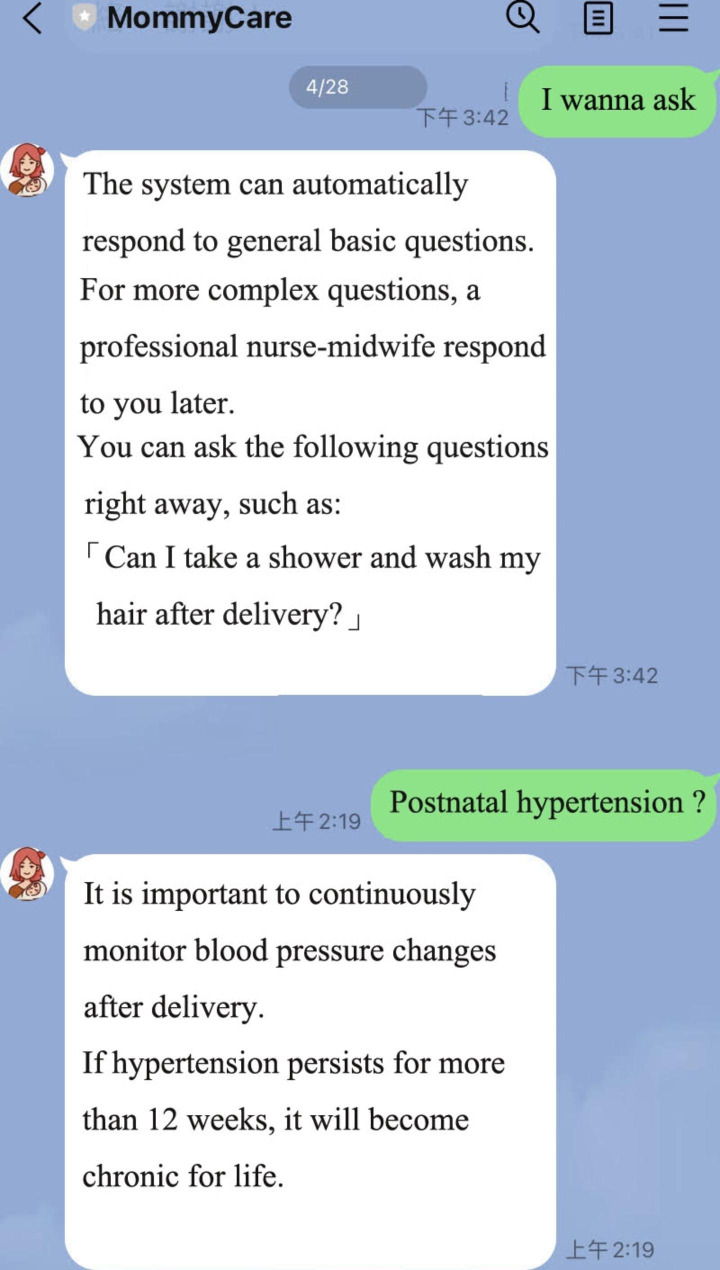
Smart chat room

The smart chat room is shown in Fig. 11. The system was designed with an intelligent chat room, where common questions from postnatal women were organized into a database. For example, queries such as “Can I take a shower and wash my hair after giving birth?”, “How soon will I have my period after birth?”, “How soon can I resume sexual intercourse after birth?”, “What are the common contraceptive methods?” and so forth. We added a lot of keywords to these questions, so that these keywords could be linked to the question answer. As long as users asked a question involving these keywords, the system would intelligently reply with the correct answer. If there was a question that could not be answered by the system, the system would first reply “This question is more complicated and will be answered by a professional nurse-midwife later.” At this point, the nurse-midwife managing the back office would think about how to answer the query, and she would then answer the user directly through a manual process.

**Fig. 12:**
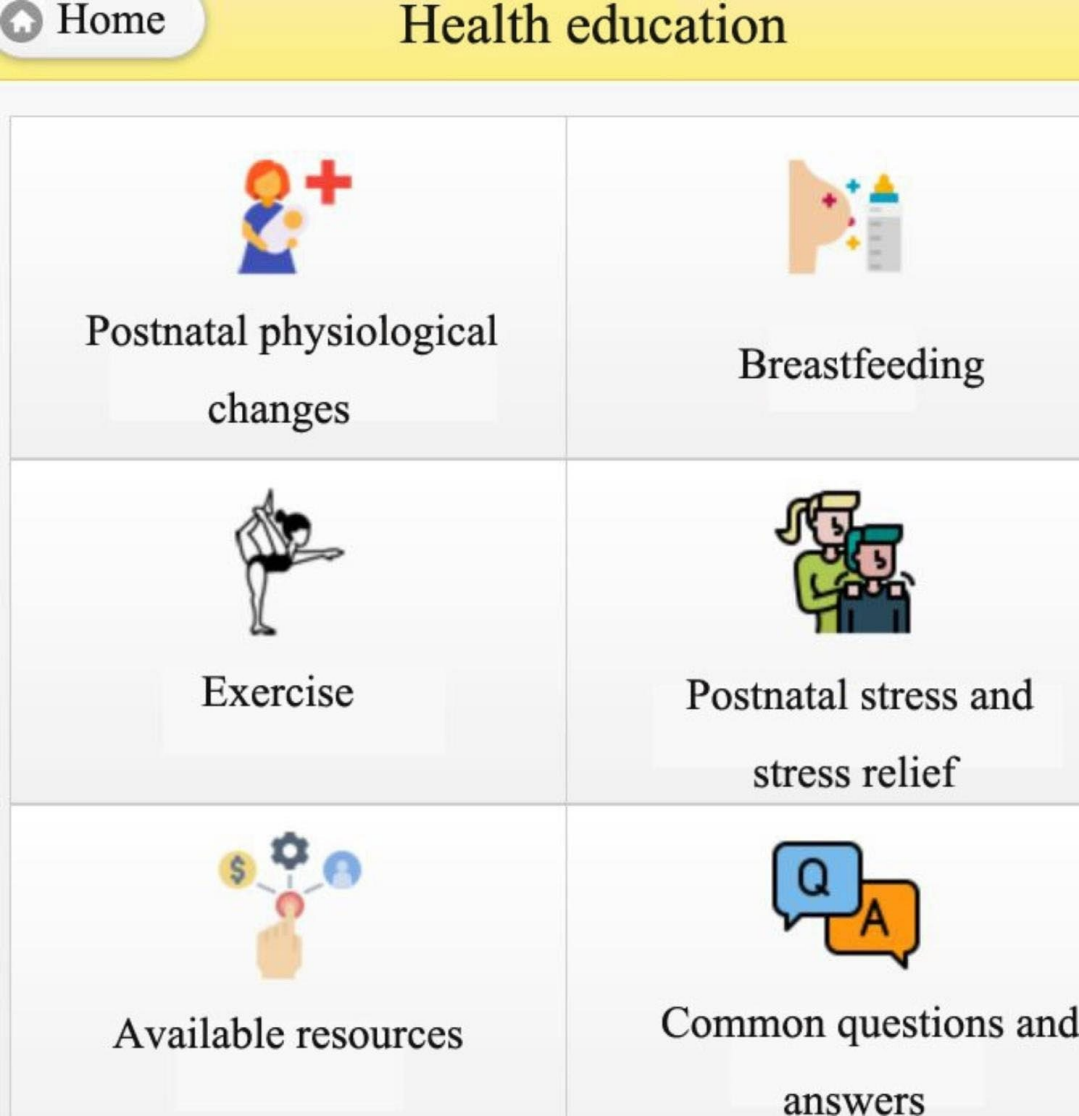
Health education information

Finally, the postnatal health education information which could be the most important professional knowledge for the postnatal women, is shown in Fig. 12. The information was organized as follows: (i) information on postnatal physiological changes, (ii) breastfeeding, (iii) postnatal exercise, (iv) postnatal stress and stress relief, (v) available resources, for example, links to websites on postnatal care and benefits given by the statements that were organized for postnatal women’s reference, and (vi) common questions and answers.

### Satisfaction evaluation

Following development, a survey of user’s satisfaction with postnatal care via the self-management eHealth application system was conducted. The satisfaction measure contained 12 items and each item was evaluated using a five-point Likert scale, namely strongly dissatisfied, dissatisfied, fair, satisfied, and strongly satisfied, represented by 1–5, respectively [21].

## Results

A total of thirty-nine women with postpartum hypertension were recruited to the survey. Convenience sampling was used to recruit maternal participants from the obstetric outpatient department at a medical center in southern Taiwan. The women adopted the system after they gave birth. Nurse-midwives at the department verbally asked women about their willingness to participate in the survey. If their answer was positive, they were recruited to the survey and were provided with access to the system. Upon logging in and registering, an online consent form first appeared for them to fill out, giving their consent to take part in the user evaluation. The system was made available to women for a three-month period. Nurse-midwives provided instructions on how to use the system and informed the users of a QR code that links them to the system.

User satisfaction involved 14-item questionnaire. The mean score for each item was greater than 4, indicating that the users were satisfied. The standard deviation ranged from 0.47 to 0.70, which suggests that there was very little fluctuation and that all respondents were satisfied with the eHealth application system. The results are presented in Table 1.

Interestingly, 94.9% (43.6 satisfied and 51.3% strongly satisfied) of the respondents stated that overall they were satisfied with the system. In addition, 92.4% (46.2% satisfied and 46.2% strongly satisfied) of the respondents stated that the systems were helpful for their postnatal health. Of all the recording tools, users reported the highest satisfaction with the blood pressure chart statistic tool (mean score 4.36). In addition, 97.4% of women reported that they would recommend this system to other postnatal women.

**Table 1:** User satisfaction with the proposed system

Topic	Mean (standard deviation)	Strongly dissatisfied (n/proportion)	Dissatisfied (n/proportion)	Fair (n/proportion)	Satisfied (n/proportion)	Strongly satisfied (n/proportion)
How do you feel about the overall system?	4.45(0.69)	0	0	2 (5.1%)	17 (43.6%)	20 (51.3%)
Did the system help your postnatal health?	4.09 (0.7)	0	0	3 (7.6%)	18 (46.2%)	18 (46.2%)
Do you think all information of this system was easy to understand?	4.27 (0.65)	0	0	2 (5.1%)	15 (38.5%)	22 (56.4%)
Can you use this system easily?	4.18 (0.60)	0	0	4 (10.3%)	16 (41.0%)	19 (48.7%)
How do you feel about the “Blood pressure& Pulse” function?	4.27 (0.65)	0	0	3 (7.6%)	14 (36.0%)	22 (56.4%)
How do you feel about the “Blood pressure chart statistic” function?	4.36 (0.50)	0	0	0	17 (43.6%)	22 (56.4%)
How do you feel about the “Weight record ” function?	4.27(0.47)	0	0	1 (2.6%)	22 (56.4%)	16 (41.0%)
How do you feel about the “Postnatal exercise” function?	4.18 (0.60)	0	0	6 (15.4%)	16 (41.0%)	17 (43.6%)
How do you feel about the “Medication record” function?	4.18 (0.60)	0	0	2 (5.1%)	25 (64.1%)	12 (30.8%)
How do you feel about the “The risk factor assessment?	4.18 (0.60)	0	0	1 (2.6%)	22 (56.4%)	16 (41.0%)
How do you feel about the “Smart chat room” function?	4.09 (0.70)	0	0	3 (7.6%)	19 (48.7%)	17 (43.6%)
How do you feel about the “Pregnant health education” function?	4.18 (0.60)	0	0	1 (2.6%)	19 (48.7%)	19 (48.7%)

## Discussion

Several studies have explored the use of self-management for postpartum hypertension. For example, Cairns et al., in a randomized controlled trial, evaluated the feasibility and effects of self-management of postpartum hypertension on blood pressure [22]. This was the first randomized evaluation of postpartum blood pressure self-management and the study suggests that such an intervention is feasible in a larger study. Control for 6 months may lead to better diastolic blood pressure, even with discontinuation of medication. In 2021, Kitt et al., reported a randomized controlled trial with long-term blood pressure monitoring [23]. Interventions to optimize blood pressure control during the postpartum period may improve blood pressure in the long term and the risk of major adverse cardiovascular events in people at increased risk of developing chronic hypertension. Kitt et al., also proposed a randomized trial called physician optimised postpartum hypertension treatment (POP-HT) [24]. The intervention can provide significant improvement for women with new-onset hypertensive postpartum at 6–9 months. This trial also used a smartphone App to assist self-management of blood pressure in the postpartum period, and medication adjustments would be monitored by the physician.

Cairns et al., reported a mixed-methods study of women’s experiences of a postpartum hypertension intervention [25]. This study used semi-structured interviews with postpartum women with medicated gestational hypertension or pre-eclampsia and included qualitative and scoring as part of a pilot randomized controlled trial. This self-management enhanced the postpartum woman’s sense of control and improved the associated anxiety.

Table 2 compares these postpartum hypertension care trials with the proposed system in this work. This proposed system possessed the four additional features to those of others (marked with a star in Table 2). These features were a multi-platform available for Windows and Mac desktops, and also feasible for iOS and Android phones, a Mobile App which was convenient to use and record on a smartphone, a self-care function with up to 4 types with line chart and pie chart graphical displays, which could make it easier for the user to understand, and a smart chat room, where postnatal women could ask questions and receive answers.

**Table 2:** Comparison of postpartum hypertension care studies

	SNAP-HT Trial [22]	Long-term Controlled Trial [23]	POP- HT Trial [24]	Mixed-methods Study [25]	Proposed System
*Multi-platform	No	No	No	No	Yes
*Mobile App	No	No	Yes	No	Yes
Specific BP Instrument	Yes	Yes	Yes	No limit	No limit
*Self-care with Graph	No	No	Yes	No	Yes
*Smart Chat Room	No	No	No	No	Yes
Trial period > 1 year	No	Yes	No	No	No

## Conclusion

In conclusion, our work proposes a humanized and individualized postpartum hypertension eHealth application system to help postnatal women adopt healthy behaviors and restore their health as soon as possible after birth. There are few eHealth applications designed for women with postpartum hypertension. The system provided humanized care, with a focus on analyzing the daily changes in postpartum hypertension, allowing doctors to more accurately and quickly review the blood pressure changes and adjust the medication in a timely manner during postnatal visits. During the postnatal recovery period, blood pressure was restored to a normal range as soon as possible to reduce the chance of women with postpartum hypertension becoming chronically hypertensive for the rest of their lives, and to reduce their chance of having to take medication to control their blood pressure long-term.

In future work, this study will extend the observation period and test the system in an experimental-control trial, inviting a larger number of postnatal women to take part to sufficiently observe the effectiveness of the system on postpartum hypertension.

## Data Availability

The datasets used and/or analyzed during the current study are available from the corresponding author on reasonable request.

## Abbreviations

PHP: Hypertext Preprocessor
